# Budesonide Analogues Preserve Stem Cell Pluripotency and Delay 3D Gastruloid Development

**DOI:** 10.3390/pharmaceutics15071897

**Published:** 2023-07-06

**Authors:** Filomena Amoroso, Eduardo Ibello, Federica Saracino, Federica Cermola, Giovanna Ponticelli, Enrica Scalera, Francesca Ricci, Gino Villetti, Gilda Cobellis, Gabriella Minchiotti, Eduardo Jorge Patriarca, Dario De Cesare, Cristina D’Aniello

**Affiliations:** 1 Stem Cell Fate Laboratory, Institute of Genetics and Biophysics “A. Buzzati Traverso”, Consiglio Nazionale delle Ricerche (CNR), 80131 Naples, Italy; 2 Department of Precision Medicine, University of Campania Luigi Vanvitelli, 80138 Naples, Italy; 3 Experimental Pharmacology & Translational Science Department, Corporate Pre-Clinical R&D, Chiesi Farmaceutici S.p.A., 43122 Parma, Italyg.villetti@chiesi.com (G.V.)

**Keywords:** drug toxicity, budesonide analogues, stem cells, pluripotency exit, 3D gastruloids

## Abstract

Small molecules that can modulate or stabilize cell–cell interactions are valuable tools for investigating the impact of collective cell behavior on various biological processes such as development/morphogenesis, tissue regeneration and cancer progression. Recently, we showed that budesonide, a glucocorticoid widely used as an anti-asthmatic drug, is a potent regulator of stem cell pluripotency. Here we tested the effect of different budesonide derivatives and identified CHD-030498 as a more effective analogue of budesonide. CHD-030498 was able to prevent stem cell pluripotency exit in different cell-based models, including embryonic stem-to-mesenchymal transition, spontaneous differentiation and 3D gastruloid development, and at lower doses compared to budesonide.

## 1. Introduction

Extrinsic cues generated by the cell–cell interactions control the cell identity/phenotype of mammalian cells, including their morphology, proliferation and motility. Of note, a reversible/dynamic process of stabilization and destabilization of intercellular interactions is crucial for normal embryo development and regeneration of adult tissues following injury. The impact of homotypic and heterotypic cell interactions on cell behavior is studied in helpful models of tissue regeneration including healing of injured skin [1,2], fibrocartilage [3], bone [4,5] and muscle [6,7,8], as well as in models of tumorigenesis as the epithelial-to-mesenchymal transition (EMT) involved in cancer progression [9,10]. Of note, the embryonic organoids generated from aggregates of pluripotent (mouse and human) stem cells, including gastruloids and blastoids, are emerging models to study collective cell behavior during early embryogenesis [11,12]. 

Growth factors and small molecules able to modulate the cell–cell physical contacts could represent useful tools to identify the underlying molecular mechanisms. In this context, glucocorticoids (GCs), stress hormones widely used for the treatment of several diseases, play critical roles during embryonic development. Of note, endogenous GCs increase during gestation, and a proper control of their levels is necessary to finely modulate the correct development of several organs [13]. Accordingly, before birth, GC levels rise to replace placenta and allow several functions including respiration and metabolism [14]. GCs act either through the glucocorticoid receptor (GR), or in an independent manner, playing a plethora of molecular effects [15]. Interestingly, recent reports shed light on a previously unrecognized activity of budesonide, a glucocorticoid that is largely used as a potent anti-asthmatic drug [16,17]. Budesonide counteracts exit from pluripotency that occurs either spontaneously upon removal of undifferentiating factors (LIF, CHIR99021, PD0325901) or is induced by L-proline supplementation (esMT) in 2D ESC culture, promoting cell–cell adhesion and reducing the phenotypic and molecular heterogeneity of ESCs. Budesonide also prevents ESC aggregates from self-organizing and generating elongated 3D gastruloids, small aggregates of pluripotent stem cells that recapitulate early stages of embryo development, a process that is still poorly characterized. Specifically, budesonide promotes compaction of 3D ESC aggregates, which eventually fail to break symmetry, at least in part by favoring cell–cell interaction and preventing cell migration. This activity of budesonide is unique among the classical glucocorticoids and it is independent of the glucocorticoid receptor (GR) [16]. 

Here, in the search for more active small molecules to investigate ESCs’ self-organization properties, we assessed the effects of structural analogues of budesonide on 2D and 3D ESC cultures and identified a new compound, named CHD-030498, which favors the maintenance of pluripotency state and prevents symmetry breaking and 3D gastruloid elongation with higher efficacy compared to budesonide.

## 2. Materials and Methods

### 2.1. Cell Cultures and Treatments

The embryonic stem cell lines: wild-type E14Tg2a (E14), shNT (control) and sh*Nr3c1* KD were used throughout this study. The E14TG2a murine embryonic stem cell line was kindly provided by Dr. Dario Acampora (from IGB-CNR, Naples, Italy). The shNT and shNr3c1 KD cell lines were previously generated from the E14TG2a parental cell line [16]. ESCs were grown in FBS/LIF in standard conditions as previously described [16,18], using high glucose DMEM (Invitrogen-Gibco, Carlsbad, CA, USA; Life Technologies, Foster City, CA, USA), 15% FBS (Euroclone S.p.A., Pero, MI, Italy), 2 mmol/L glutamine and penicillin/streptomycin (100 U/mL; Invitrogen-Gibco, Carlsbad, CA, USA). All the experiments were performed between the 10th and the 25th cell passage. For all cell lines, Mycoplasma-free state is routinely (twice/year) tested by PCR-based assay. Budesonide, fluticasone and BA-GCs compounds (provided by CHIESI Farmaceutici, Parma, Italy) were dissolved in DMSO at a concentration of 10 mM and used at a concentration range of between 10^−5^ and 10^−10^ M.

### 2.2. Cytotoxicity, Proliferation and Apoptosis Assays

To assess the cytotoxic effects of BA-GCs, ESCs were plated at 1 × 10^4^ cells/cm^2^, in triplicate, on gelatin-coated 96-multiwell plates (Corning Inc., Midland, NC, USA) ± increasing concentrations (10^−10^, 10^−9^, 10^−8^, 10^−7^, 10^−6^, 10^−5^ M) of the compounds or with the solvent (DMSO) alone as control. Halofuginone (HF; nM concentrations) was used as additional control [19]. After 24 h in culture, cells were incubated with CCK-8 (10 μL; Dojindo Laboratories, Kumamoto, Japan) for 24 h, as previously described [17]. The absorbance, which is directly proportional to the number of living cells, was measured at 450 nm using the spectrophotometer Synergy H1 Microplate Reader (BioTek, Agilent Technologies, Santa Clara, CA, USA). 

For cell proliferation, we used the 5-ethynyl-2′-deoxyuridine (EdU; Invitrogen-Gibco, Carlsbad, CA, USA) assay. Briefly, 1 × 10^4^ ESCs/cm^2^ were plated on gelatin-coated plates (Corning), and treated with the different compounds. Two or three concentrations among the highest (10^−5^, 10^−6^ and 10^−7^ M) were used for each compound. CHD-030498 was used at 5, 2.5, 1 and 0.1 μM, since at 10 μM a toxic effect was shown. DMSO was used as a control. After 24 h in culture, cells were incubated with EdU (10 μM) overnight. ESCs were then detached with trypsin and the EdU staining protocol was performed, following the manufacturer’s instructions. Briefly, cells were fixed for 10 min at room temperature (RT). After washing and permeabilization with a saponin-based buffer for 10 min at RT, cells were stained with a cocktail solution (EdU kit), containing the 488 fluorophore. After an incubation period of 30 min in the dark, cells were washed and analyzed by FACS (Aria II cell sorter Becton Dickinson). One well of untreated cells (UT) was left without EdU treatment as negative control.

Apoptosis was analyzed using annexin V/propidium iodide (PI) staining (Dojindo Laboratories, Kumamoto, Japan). Briefly, 1 × 10^4^ ESCs/cm^2^ were seeded on gelatin-coated plates (Corning) and treated with the different compounds (10^−5^, 10^−6^ and 10^−7^ M), as above. ESCs were treated ± the solvent (DMSO) as control. After 48 h in culture, both adherent and suspension cells were collected following standard procedures, and apoptosis was measured by annexin V/propidium iodide (PI) staining, following the manufacturer’s instructions. Briefly, cells were counted and resuspended at final concentration of 1 × 10^6^ cells /mL in annexin V binding solution, containing both annexin V and PI, for 15 min at RT in the dark. DMSO-treated cells that were not stained with annexin V/PI were used as a negative control. Then, the staining solution was diluted 1:5 and the samples were analyzed by FACS (Canto, Becton Dickinson, Franklin Lakes, NJ, USA).

### 2.3. esMT/MesT

Embryonic stem cell to mesenchymal-like transition (esMT) was performed as previously described [17,20]. Briefly, ESCs were plated at low density (300 cells/cm^2^) on gelatin-coated plates and treated ± proline (250 μM), ± the compounds supplemented at different final concentrations (from 10^−11^ to 10^−5^ M). Budesonide and fluticasone were used at 10 μM as a positive and a negative control, respectively. The dose-dependent effect of CHD-030498 was analyzed in detail at 10, 7.5, 5, 2.5 and 1 μM. For mesenchymal-like to embryonic stem cell transition (MesT), freshly generated proline-induced mesenchymal-like cells (PiCs) were plated at low density (300 cells/cm^2^) and treated ± proline (250 μM), as controls, and ± the compounds, at concentrations ranging from 10^−5^ to 10^−7^ M. At day 5 the colonies resulting from either esMT or MesT were fixed and stained with a solution of PBS1x/6% glutaraldehyde/0.15% crystal violet, and the frequency of domed versus flat colonies were scored as a quantitative readout of the phenotypic transition as previously described [17,20].

### 2.4. Pluripotency Exit

ESCs were cultured in FBS/2i/LIF medium for 3 days and then shifted to either FBS alone, FBS + budesonide (10 μM), fluticasone (10 μM), CHD-030498 (10, 5, 2.5 and 1 μM) or maintained in FBS/2i/LIF as a control for an additional 4 days. The resulting colonies were fixed and stained with a solution of PBS1x/6% glutaraldehyde/0.15% crystal violet and the frequency of flat colonies was scored [16,17].

### 2.5. Gastruloid Formation Assay 

Gastruloids were generated as described [16,18,21]. ESCs were seeded at 2.5–3.0 × 10^2^ cells/well. CHIR99021 (3 μM) was added between 48 and 72 h. From 72 h onwards, the medium was refreshed every day. Budesonide (1 and 10 μM), CHD032201 (1 and 10 μM) and CHD-030498 (10, 1, 0.5, 0.25 and 0.125 μM) were added either during the first 48 h or for 120 h. Gastruloids were imaged using EVOS. The aggregate’s diameter at 48 h was analyzed using Image J 1.46r software (https://imagej.nih.gov/ij/). The elongation index was calculated using ImageJ-Fiji (BIOP plugin).

### 2.6. RNA Extraction and Real Time PCR

To perform total RNA extraction, we used Trizol (Life Technologies). Total RNAs were reverse transcribed with the High-Capacity cDNA Reverse Transcription kit (Applied Biosystems, Waltham, MA, USA). To analyze gene expression, we performed real-time PCR using the SYBR Green PCR master mix (FluoCycle IITM SYBR, Euroclone S.p.A., Pero, MI, Italy) and a BioRad CFX qPCR machine. The primer sequences are reported in Table 1 below.

### 2.7. Immunofluorescence 

To perform immunofluorescence on gastruloids and spheroids we used the protocol previously described [21,22]. Briefly, gastruloids were washed (3×, 10 min) at RT with PBS, then with PBS/10% FBS/0.5% Triton X-PBSFT (3×, 10 min) and finally with PBSFT (1 h, 4 °C). Gastruloids and spheroids were then incubated with the following specific antibodies (48 h, at 4 °C) on a low-speed orbital rocker: E-cadherin (1:250, Takara, Saint-Germain-en-Laye, France); Oct4 (1:100, Santa Cruz Biotechnology, Dallas, TX, USA); Nanog (1:400, Cell Signaling, Danvers, MA, USA); Cdx2 (1:100, Cell Signaling, Danvers, MA, USA); Bra (1:500, Cell Signaling, Danvers, MA, USA) and Sox2 (1:100, Cell Signaling, Danvers, MA, USA). Images were obtained using a confocal Nikon A1 microscope. Then NIS Element C (Nikon, Tokyo, Japan) software was used for image acquisition/elaboration.

### 2.8. Statistical Analysis

The number of independent experiments is specified as “*n*”, and the total sample size is indicated in each figure. Results are represented as mean ± standard deviation (SD) or standard error of the mean (SEM), or as a boxplot/dot plot indicating the minimum first quartile, median, third quartile and maximum. Comparisons were performed using two-tailed paired Student’s *t*-test. Differences were considered statistically significant when *p* values were ≤0.05. Graphs and boxplots were created using Microsoft Excel or RStudio software (Version 1.1.463—© 2009–2018 RStudio, Inc.) available at https://www.rstudio.com/.

## 3. Results

### 3.1. Effect of Budesonide-Analogue Glucocorticoids (BA-GCs) on ESC Proliferation

**Figure 1:**
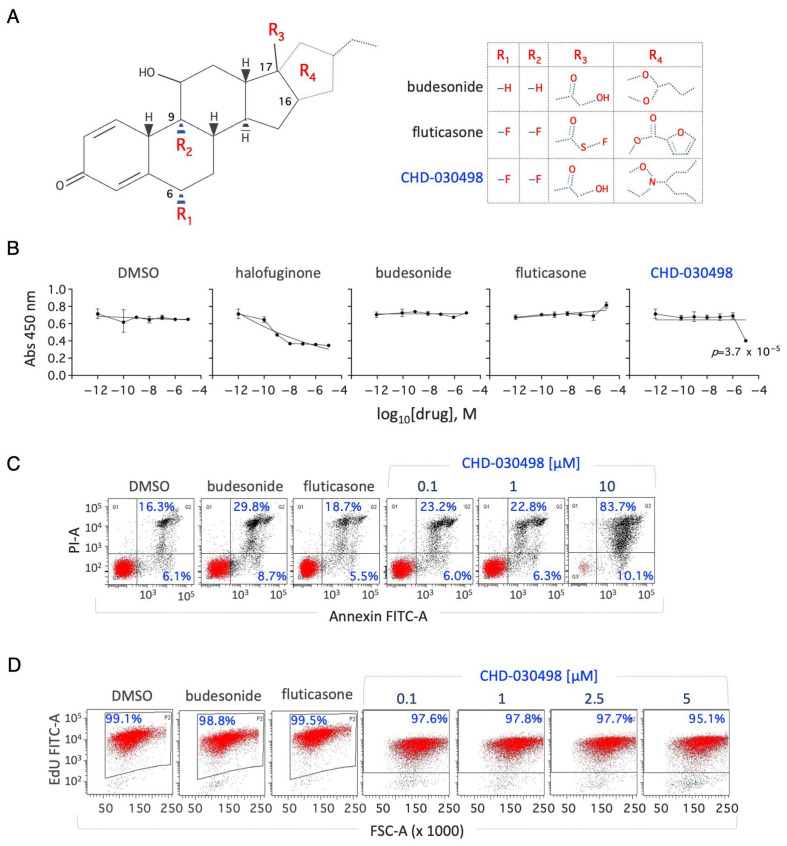
Effect of CHD-030498 on ESC proliferation. (**A**) Basic 4-ring steroid backbone of glucocorticoids is shown. Residual groups (from R1 to R4) for budesonide, fluticasone and CHD-030498 are represented. (**B**) Cell survival was analyzed using the CCK-8 cytotoxicity assay, after 48 h of treatment. Compounds were tested in a large concentration range (from 10^−10^ to 10^−5^ M). Halofuginone was used as a positive (toxic compound) control. Data are shown as mean ± SD (*n* = 3; *p* ≤ 0.01). (**C**) FACS analysis of apoptotic cells. Representative dot plot of annexin V/PI staining in ESCs treated with DMSO, budesonide (10 μM), fluticasone (10 μM) and CHD-030498 (0.1, 1 and 10 μM). The fraction (%) of annexin V^+^ (bottom right) and annexin V^+^/PI^+^ (up) cells are indicated. Annexin V is conjugated with FITC. (**D**) Dose–response effect of CHD-030498 on ESC proliferation. Representative dot plot of EdU incorporation in ESCs treated with DMSO, budesonide (10 μM), fluticasone (10 μM) and CHD-030498 (0.1, 1, 2.5 and 5 μM). The fraction (%) of cells that incorporated EdU is shown.

To search for budesonide analogues, we tested a few compounds having the basic 4-ring steroid backbone and different residues in positions 6, 9, 16 and 17 (Figure 1A). For instance, compound CHD-030498 (MW 521, 65) contains a fluor atom, instead of hydrogen, in positions 6 (R1) and 9 (R2), such as fluticasone. Moreover, it contains a 17-(2-hydroxyacetyl) residue in position 17 (R3), and a pentacyclo structure involving positions 16 and 17 (R4), such as budesonide (Figure 1A). Thus, CHD-030498 displays structural features of both fluticasone and budesonide. We first tested the effect of the selected compounds, hereafter named budesonide-analogue glucocorticoids (BA-GCs), on embryonic stem cells (ESCs) by using a CCK-8-sensitive colorimetric assay (Figure 1B and Supplementary Figure S1A). Our analysis revealed that: (i) halofuginone (HF), a potent inhibitor of prolyl-tRNA synthetase used as a positive control [19], reduced ESC survival in a dose-dependent manner; (ii) budesonide did not exert any toxic effects in the same concentration range (1 nM to 10 μM) as fluticasone (Figure 1B); (iii) CHD-030498 was toxic at the highest concentration (10 μM Figure 1B); (iv) none of the other BA-GCs assayed, including CHD-030614, CHD-032201, CHD-031019, CHD-026409, CHF6162.00 and CHD6183.00, affected ESCs’ viability/proliferation at any concentrations (Supplementary Figure S1A). 

To support these findings, we performed annexin V/PI apoptosis assay, which allows distinguishing live (annexin V^−^/PI^−^) from dead cells, and discriminating early (annexin V^+^/PI^−^) and late (annexin V^+^/PI^+^) apoptotic cells or necrotic cells (annexin V^−^/PI^+^). Quantification of the annexin V^±^/PI^±^ cell fraction revealed that at 1 μM, budesonide, fluticasone (Figure 1C) and most of the BA-GCs assayed (Supplementary Figure S1B) did not induce apoptosis and/or necrosis in ESCs (Figure 1C and Supplementary Figure S1B). Instead, at 10 μM some BA-GCs, including CHD-030614, CHD-031019, CHD-026409, CHF6162.00 and CHD6183.00, induced a two-fold increase in the fraction of apoptotic cells (Supplementary Figure S1B). The fraction of annexin V^+^/PI^+^ cells increased significantly from ~20% to ~80%, in the presence of CHD-030498 at 1 and 10 μM, respectively (Figure 1C). A cell counting analysis revealed that 10 μM CHD-03049 increased the fraction of trypan blue-positive (dead) cells. 

To further validate these data, we assessed the impact of BA-GCs on ESC proliferation by 5-ethynyl-2′-deoxyuridine (EdU) incorporation. The fraction of EdU^+^ (proliferating) cells was comparable (ranging around 98–99%) among the different BA-GCs tested (Figure 1D and Supplementary Figure S1C), confirming the absence of any toxic effects. Of note, CHD-030498 did not significantly alter ESCs proliferation in a wide concentration range (100 nM to 5 μM) (Figure 1D). 

### 3.2. BA-GCs Prevent Embryonic Stem Cell-to-Mesenchymal Transition (esMT) Induction

**Figure 2:**
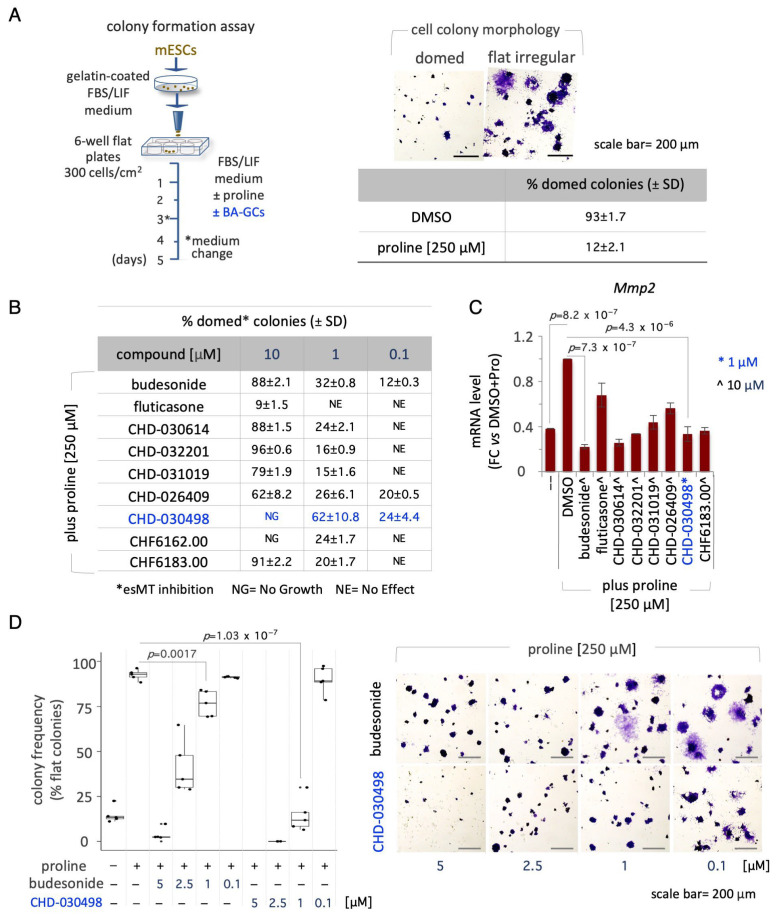
Effect of BA-GCs on embryonic stem cell-to-mesenchymal transition (esMT). (**A**) Schematic representation of the experimental procedure (left). Representative photomicrographs of domed/regular- and flat/irregular-shaped cell colonies (top right) and quantification of the fraction (%) of domed colonies generated from ESCs treated ± proline (250 μM) (bottom right). (**B**) Table showing the fraction (%) of domed colonies (esMT inhibition) generated from ESCs treated + proline (250 μM) ± BA-GCs supplemented at the indicated concentrations. Budesonide and fluticasone were used, respectively, as a positive (esMT inhibitor) and negative (inactive) control. Data are shown as mean ± SD (*n* = 3). (**C**) Effect of BA-GCs on the expression of matrix metallopeptidase 2 (*Mmp2*) gene. qPCR analysis was performed using total RNA extracted after 48 h of treatment with proline (250 μM) ± BA-GCs (10 μM). CHD-030498 was used at 1 μM. Values are expressed as fold-change versus DMSO-treated ESCs, after normalization to *Gapdh* and are mean ± SEM (*n* = 3). (**D**) Dose-dependent effect of CHD-030498 on esMT. Representative photomicrographs (right) and quantification (left) of the colonies generated from ESCs treated ± proline (250 μM), plus CHD-030498 at 0.1, 1, 2.5 and 5 μM. Data are shown as mean ± SD (*n* = 3).

We then tested the ability of BA-GCs to counteract exit from pluripotency in different 2D culture conditions. First, we analyzed the ability of BA-GCs to prevent the transition from naïve to early primed pluripotency induced by proline supplementation (esMT) [20]. Briefly, we performed colony formation assays (CFAs) by plating ESCs at low density on gelatin-coated plates ± proline (250 μM) ± BA-GCs (0.01 nM to 10 μM) (Figure 2A). Budesonide and fluticasone were used as positive and negative controls, respectively [16,17]. Following five days of culture, the cell colonies were fixed, stained and imaged; esMT was evaluated by measuring the fraction of round domed (naïve embryonic) versus irregular flat (mesenchymal-like) colonies (Figure 2A). The results showed that: (i) budesonide, unlike fluticasone, prevented the acquisition of mesenchymal/flat morphology associated with esMT (Figure 2B); (ii) at 10 μM, CHD-030614, CHD-032201, CHD-031019, CHD-026409 and CHF6183.00 inhibited esMT similarly to budesonide (Figure 2B); (iii) at 1 μM, most of the compounds were inactive, whereas CHD-030498 exerted a stronger inhibitory effect, showing ~60% of domed colonies. In line with the results obtained with the CCK-8 assay, no colonies were detected in the presence of CHD-030498 at 10 μM (Figure 2B). Furthermore, we analyzed the expression of the *Mmp2* (matrix metallopeptidase 2) mesenchymal marker known to be induced by proline [20]. Of note, *Mmp2* upregulation was reduced to a similar extent in the presence of 10 μM budesonide, CHD-030614, CHD-032201, CHD-031019, CHD-026409 and CHF6183.00 (Figure 2C). Finally, we performed a dose-dependent assay and confirmed that while CHD-030498 activity was comparable in the 5 to 1 μM range, budesonide activity progressively decreased from 5 to 1 μM (Figure 2D).

We then assessed the ability of BA-GCs to drive the reverse process of esMT, named mesenchymal-to-embryonic transition (MesT). Freshly generated proline-induced cells were dissociated and replated at low density (300 cells/cm^2^) in undifferentiation medium ± proline (250 μM), ± BA-GCs added at concentrations ranging from 0.1 to 10 μM (Supplementary Figure S2A). At day four, the frequency of reverted (domed-shaped) colonies was scored as a parameter of MesT transition. Proline addition reduced the fraction of domed colonies compared to untreated cells (Supplementary Figure S2A), whereas budesonide (10 μM) efficiently counteracted the acquisition of the mesenchymal phenotype, as previously reported [17] (Supplementary Figure S2B). CHD-030498 reverted the mesenchymal phenotype at 1 μM, inducing the formation of domed colonies similarly to budesonide (~70%) at 10 μM (Supplementary Figure S2B).

These results suggest that CHD-030498 counteracts the acquisition of the mesenchymal phenotype with a higher potency than budesonide and the other analogues.

### 3.3. EsMT Inhibitory Activity of BA-GCs Is Independent of the Glucocorticoid Receptor (GR)

We recently showed that the budesonide inhibitory effect on esMT is independent of the glucocorticoid receptor (GR) [16]. We thus asked whether GR was involved in BA-GC-mediated inhibition of esMT. To address this question, we used Nuclear Receptor Subfamily 3 Group C Member 1 (*Nr3c1*, GR) knocked down (KD) ESC lines [16]. We first tested the ability of *Nr3c1* KD ESCs to proliferate and eventually to undergo esMT. The analysis of the EdU incorporation showed comparable EdU intensity means in both the control (shNT, not targeting) and sh*Nr3c1* ESC lines in the absence of proline (Supplementary Figure S3A). Moreover, proline supplementation induced proliferation of both shNT and sh*Nr3c1*#2 ESCs to the same extent, as indicated by increased EdU incorporation (Supplementary Figure S3A); thus, suggesting that *Nr3c1* KD does not affect ESC proliferation. 

**Figure 3:**
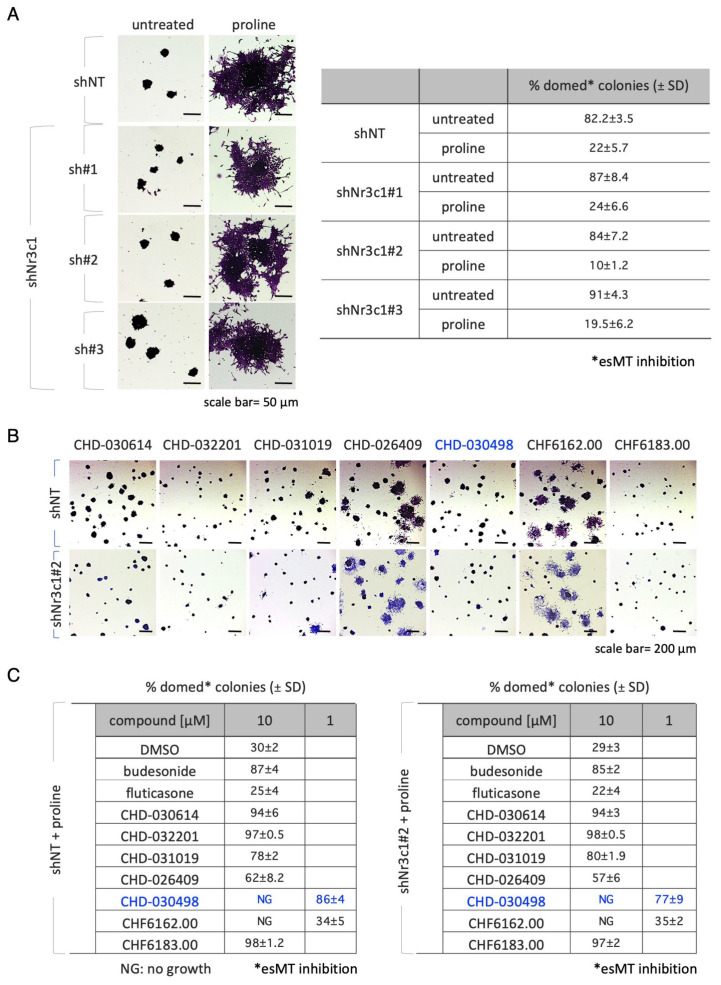
Effect of glucocorticoid receptor on BA-GCs-mediated modulation of esMT. (**A**) Representative photomicrographs (left) of cell colonies generated from shNT (non-targeting control) and shNr3c1#2 (GR knock-down) ESCs under esMT-inducing condition (supplemental proline, 500 μM). Table showing the fraction (%) of domed-shaped cell colonies (esMT inhibition) (right). Scale bar, 50 μm. (**B**) Representative photomicrographs of colonies generated from shNT and shNr3c1#2 ESCs treated ± proline (500 μM) ± BA-GCs (10 μM), and stained with crystal violet. CHD-030498 was used at 1 μM. (**C**) Tables showing the fraction (%) of domed colonies (esMT inhibition), in the different conditions (shNT, bottom left; sh*Nr3c1*#2, bottom right). Budesonide and fluticasone were used as positive (esMT inhibitor) and negative (inactive) controls. The different effects of CHD-030498 at 10 (toxic) and 1 μM are highlighted in blue. Data are shown as mean ± SD (*n* = 3). Scale bar, 200 μm.

We then analyzed the effect of *Nr3c1* KD on esMT. The quantification analysis (domed versus flat shaped colonies) showed that the fraction of domed colonies was comparable in the sh*Nr3c1*#1–3 cell lines and shNT control cells (Figure 3A) and thus indicating that *Nr3c1* KD ESCs undergo esMT. These results were further confirmed at molecular level by qPCR expression analysis of the mesenchymal markers *Fgf5*, *N-Cadherin* and *Brachyury/T*. Specifically, we found that expression of all the genes analyzed was strongly and similarly induced in both shNT and sh*Nr3c1*#2 ESCs upon proline supplementation (Supplementary Figure S3B). 

Based on the evidence that a high levels of GR expression is dispensable for esMT, we tested budesonide and BA-GCs on *Nr3c1* KD ESCs during the esMT transition. Thus, the control shNT and sh*Nr3c1*#2 cell line were plated at low density (300 cells/cm^2^) on gelatin-coated plates and treated ± proline (500 μM), ± budesonide and fluticasone as controls and ± BA-GCs at the active concentrations. As expected, budesonide, but not fluticasone, inhibited proline-dependent acquisition of mesenchymal/flat morphology in sh*Nr3c1* ESCs as efficiently as in the shNT control (Figure 3B). BA-GCs used at 10 μM, with the exception of CHF6162.00, also inhibited esMT in the absence of GR (Figure 3C). Of note, in the presence of 1 μM CHD-030498 the sh*Nr3c1*#2 cell lines were unable to undergo esMT. 

These findings suggest that CHD-030498 is a potent BA-GC, able to inhibit esMT in a GR-independent manner, remarkably at a concentration 10 times lower than all the other compounds assayed.

### 3.4. BA-GCs Counteract Exit from Pluripotency in Cell Colonies and Spheroids

**Figure 4:**
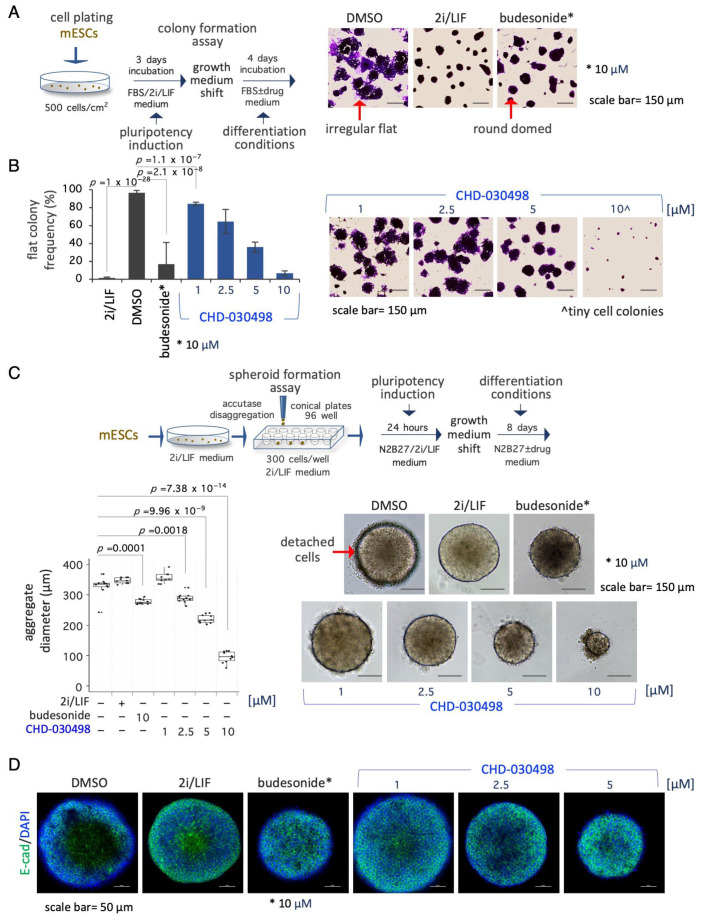
Effect of BA-GCs on pluripotency exit. (**A**) Schematic representation of the experimental procedure (left). ESCs were plated (500 cells/cm^2^) in DMEM/FBS/2i/LIF medium for 3 days. The resulting domed-shaped colonies were shifted to DMEM/FBS medium supplemented with DMSO, 2i/LIF, fluticasone or budesonide. After 4 days of incubation the cell colonies were imaged or fixed and stained with crystal-violet (right). Scale bar, 150 μm. In the absence of undifferentiating factors, such as the 2i/LIF mix, ESCs proliferate generating irregular flat- instead of round domed-shaped cell colonies. (**B**) Dose–response activity of CHD-030498 on the morphological transition associated with pluripotency exit. Histogram showing the fraction (%) of flat-shaped cell colonies (left) generated in the presence of budesonide (10 μM, used as a positive control), or CHD-030498 (from 1 to 10 μM). Data are mean ± SD (*n* = 3; 10 fields/condition). Representative images of crystal violet-stained cell colonies (right). Scale bar, 150 μm. (**C**) Schematic representation of the experimental procedure (top). Pluripotent ESCs (2i/LIF) were FACS sorted (300 cells/well) on 96-well ultra-low conical plates and incubated in N2B27 ± CHD-030498 (from 1 to 10 μM). After 7 days of incubation the resulting spheroids were imaged and measured. Boxplot diagrams of aggregate diameter at day 7 (left; 10 spheroids/condition), and representative bright-field images (right) of spheroids treated with DMSO (control), or CHD-030498 used at the indicated concentration. Scale bar, 150 μm. (**D**) Immunofluorescence analysis of E-cadherin (E-cad) expression. Representative confocal images of 8-day-old spheroids, generated as described in (**C**). Nuclei were counterstained with DAPI (blue). Scale bar, 50 μm.

We then tested the effect of the most potent BA-GC, i.e., CHD-030498, on spontaneous ESC differentiation induced by 2i/LIF withdrawal. Thus, ESCs were first cultured in FBS/2i/LIF for three days and then shifted to a medium containing FBS ± CHD-030498 or budesonide or kept in FBS/2i/LIF as controls. After a further four days of culture the colony morphology was analyzed (Figure 4A). As previously described [16], while FBS/2i/LIF and budesonide-treated ESC colonies maintained a domed-shaped and regular morphology, untreated cell colonies undergo a morphological transition, generating flat-irregular colonies (96.6% ± 2.4) (Figure 4A,B). Differently from budesonide, fluticasone and CHD-032201 were unable to counteract spontaneous differentiation (Supplementary Figure S4A). Similar to budesonide, CHD-030498-treated ESC colonies maintained the domed-shaped morphology in a dose-dependent manner. Of note, the generation of differentiated flat-shaped cell colonies was reduced from 84.4 ± 1.8, to 64.4 ± 13.6 and 35.9 ± 5.6% at 1, 2.5, and 5 μM CHD-030498, respectively (Figure 4B). At 10 μM CHD-030498, only small colonies were formed (Figure 4B), consistent with the observed reduction of ESC proliferation and induction of apoptosis at this concentration (see Figure 1). Our results suggest that CHD-030498 is able to counteract exit from pluripotency induced by removal of 2i/LIF by promoting the generation of highly compacted cell colonies, and inducing the stabilization of cell–cell interactions.

We finally assessed the ability of CHD-030498 to counteract spontaneous differentiation in 3D culture conditions as well (Figure 4C). To this aim, naïve pluripotent ESCs were seeded by FACS in ultra-low attachment U-round bottom plates (300 cells/well, 96-well plate) and allowed to aggregate in N2B27 medium supplemented with 2i/LIF for 24 h, to generate highly compacted floating spheroids. The spheroids were then shifted to different conditions including DMSO alone (differentiating conditions), 2i/LIF (pluripotency maintenance), budesonide (10 μM) or CHD-030498 used at different concentrations from 1 to 10 μM (Figure 4C and Supplementary Figure S4C). Similar to budesonide, CHD-030498-treated ESC spheroids maintained a domed-shaped morphology and a reduced dimension, in a dose dependent manner. Moreover, the resulting spheroids (eight-day-old) were highly compacted and similar to the 2i plus LIF condition, as shown by E-cadherin staining (Figure 4D). In contrast, in the absence of inhibitors/cytokines (2i/LIF), untreated spheroids (DMSO) showed an irregular/loose structure, suggesting a differentiation process (Figure 4D). Our results suggest that CHD-030498 is able to counteract exit from pluripotency in 3D spheroids, induced by removal of 2i/LIF. 

### 3.5. BA-GCs Prevent Symmetry Breaking and Gastruloid Elongation

**Figure 5:**
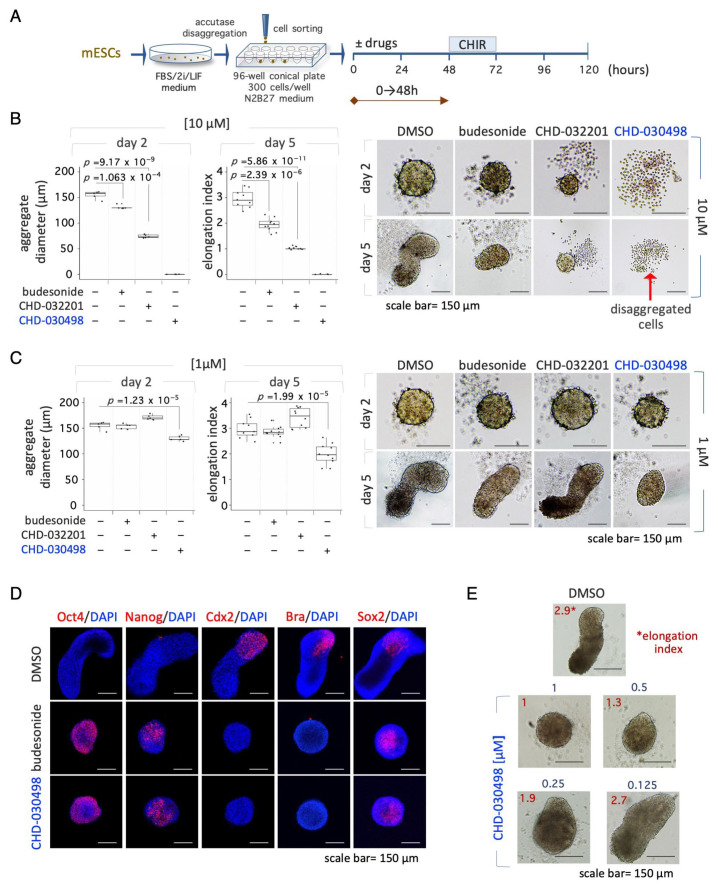
Effect of BA-GCs on gastruloid development. (**A**) Schematic representation of the experimental design. Pluripotent ESCs (2i/LIF) were FACS sorted (300 cells/well) on 96-well ultra-low conical plates and incubated in N2B27 ± CHD-032201 or CHD-030498 (10 and 1 μM). (**B**,**C**) Boxplot diagrams of aggregate diameter at day 2 (left; 10 aggregates/condition), and of gastruloid elongation index (middle) and representative bright-field images (right) of gastruloids treated with DMSO (control), CHD-032201 or CHD-030498 at 10 (**B**) and 1 μM (**C**) (10 gastruloids/condition). Scale bar, 150 μm. (**D**) Immunofluorescence analysis of pluripotency (Oct4, Nanog) and differentiation (Cdx2, brachyury, Sox2) marker expression. Representative confocal images of 5-day-old aggregates/gastruloids, untreated or treated with budesonide (10 μM, positive control) or with CHD-030498 (1 μM). Nuclei were counterstained with DAPI (blue). Scale bar, 150 μm. (**E**) Dose-dependent effect of CHD-030498, added at 1, 0.5, 0.25 and 0.125 μM, from T0 to 120 h. The elongation index is shown (10 gastruloids/condition). Scale bar, 150 μm.

We then evaluated the effect of CHD-030498 on gastruloid development. To this end, ESCs cultured in naïve pluripotency-inducing conditions (2i/LIF) were FACS-sorted in ultra-low attachment U-round bottom plates (300 cells/well); budesonide, CHD-032201 and CHD-030498 were added at 1 μM and 10 μM within the 0–48 h time window (Figure 5A). At days two and five the resulting organoids were imaged, and the diameter was measured and the elongation index (EI) was calculated. As previously shown, 10 μM budesonide reduced the diameter of two-day-old aggregates compared to control [16]. By day five, control aggregates efficiently elongated, whereas budesonide-treated aggregates failed to develop into fully elongated gastruloids (EI = ~1.5–2) (Figure 5B) [16]. Of note, 10 μM CHD-032201 allowed the generation of small-sized spheroids, which maintained a spheroidal shape even after five days of incubation (Figure 5B). Finally, CHD-030498 and CHD-032201 at 10 μM showed toxic effects on both days two and five (Figure 5B), according to the toxic effect shown above, for CHD-030498, on ESC culture (Figure 1).

Since the formation of spheroids with a precise diameter (around 150–180 μm) is an essential step for the proper development of elongated gastruloids [21], we analyzed the effect of a lower concentration (1 μM) of BA-GCs on ESC aggregation. Similar to 1 µM budesonide, ESCs treated with 1 μM CHD-032201 were able to form properly shaped and sized aggregates, which eventually elongated into 3D gastruloids (Figure 5C). In contrast, 1 μM CHD-030498 induced the generation of small aggregates, which failed to elongate, showing an effect similar to that of 10 μM budesonide (Figure 5B,C). Moreover, CHD-030498- treated aggregates (1 μM) did not express the brachyury (Bra, T) and Cdx2 developmental markers, and stained positive for the Oct4 and Nanog pluripotency markers, similar to that observed with budesonide (10 μM) (Figure 5D). We then performed a dose-dependent (ranging from 1 to 0.1 μM) gastruloid formation assay in the presence of CHD-030498 throughout the experiment (0–120 h) (Supplementary Figure S5). Of note, the EI significantly decreased when the concentration of CHD-030498 increased, reaching values of 2.7, 1.9, 1.3 and 1 at 0.125, 0.25, 0.5 and 1 μM, respectively (Figure 5E and Supplementary Figure S5C). At 0.125 μM CHD-030498 the EI was comparable to the control (Supplementary Figure S5C) and, accordingly, the elongated gastruloids correctly expressed Bra (Supplementary Figure S5D).

In conclusion, our results suggest that only CHD-030498 affects gastruloid development, acting similarly to budesonide, but at a lower concentration.

## 4. Discussion

In this study, we analyzed the effects of a small set of synthetic glucocorticoids (budesonide analogue glucocorticoids; BA-GCs), using 2D and 3D stem cell-based assays, as an in vitro paradigm of pluripotency exit. 

Over the last years, the use of small molecules has largely increased, as a complementary approach to classical genetics, to investigate several biological functions. In particular, small molecules have represented useful tools to study stem cell biology and explore the underlying mechanisms of stem cell self-organization and cell–cell communication, by which initially homogeneous populations of cells break symmetry and undergo in vivo-like pattern formation and morphogenesis. These mechanisms are attracting great interest in the emerging field of synthetic embryology, and are still poorly defined.

Here we compared the activity of the BA-GCs on ESCs to that recently described for budesonide [16]. Our results indicate that most of the BA-GCs counteract exit from pluripotency in different in vitro models, including spontaneous differentiation (LIF/2i withdrawal) and esMT triggered by proline supplementation, at levels comparable to that of budesonide. Interestingly, among the BA-GCs, one compound, named CHD-030498, shows increased activity, being able to inhibit the naïve to primed transition induced by proline at a concentration ten times lower than budesonide. The morphological changes are associated with variations at a molecular level. Of note, *Mmp-2*, a gene induced by proline, is inhibited by the BA-GCs. In particular, CHD-030498 reduces *Mmp-2* levels similarly to budesonide, but at a lower concentration. CHD-030498 also promoted reversed transition, facilitating the formation of pluripotent/domed colonies in the presence of proline, and showing a higher efficacy than budesonide and the other BA-GCs, acting at 1 μM. Of note, while none of the tested compounds shows a toxic effect on ESCs, only CHD-030498 reduces ESC survival and induces apoptosis at higher concentration. However, when reducing its concentration (from 5 to 1 μM), proliferation is not altered anymore. 

GCs act by binding to the glucocorticoid receptor and can either exert transcriptional activity in the nucleus, or repression in the cytoplasm [15]. Beside the well-known GR-dependent effects, GCs can also induce GR-independent (non-genomic) effects, which are still not completely elucidated [15]. Budesonide has recently been shown to inhibit exit from pluripotency in 2D ESC cultures in a glucocorticoid-independent manner [16]. Our findings show that BA-GCs’ ability to counteract pluripotency exit is independent from the GR, suggesting that, similarly to what is observed for budesonide, other targets may be involved. In support of our conclusions, fluticasone and the *Nr3c1* knock-down do not affect pluripotency exit, as previously shown [16].

Budesonide has also been shown to inhibit exit from pluripotency in 3D gastruloids, promoting cell–cell adhesion and compaction. Similarly, CHD-030498 maintains round and compacted floating aggregates and avoids pluripotency exit and gastruloid development. Of note, as for 2D cultures, CHD-030498 inhibits pluripotency exit in 3D gastruloids at a concentration ten times lower compared to budesonide. Interestingly, another BA-GC, namely, CHD-032201, is active in 2D cultures, but not in 3D cell aggregates, highlighting the differences between 2D and 3D cultures and the importance of comparing drug effects in both conditions. 

CHD-030498-dependent inhibition of pluripotency exit is supported by the persistent expression of the Oct4 and Nanog pluripotency markers in 3D gastruloids/aggregates and the absence of the Cdx2 and Bra differentiation markers. Of note, CHD-030498-dependent molecular effects also occur at a concentration ten times lower than budesonide. The molecular mechanisms underlying the establishment of stem cellular identity and behavior during development are still largely unknown. However, the study of the molecular effects of BA-GCs, such as CHD-030498, might be helpful to elucidate how cell–cell interactions influence pluripotency exit and differentiation. 

Budesonide belongs to the corticosteroid class of medications, commonly used to treat inflammatory diseases, including asthma, Crohn disease and ulcerative colitis. In spite of their beneficial effects, collateral unwanted reactions are often observed following high doses and prolonged corticosteroid treatment. These adverse effects have been mainly ascribed to GR-independent ‘nongenomic’ effects, which hinge on nonspecific interactions of steroidal molecules with cellular membranes. GCs play critical roles during development, contributing not only to the correct differentiation of several organs, but also performing metabolic functions. However, if exogenously administered, GCs can be either beneficial or detrimental for embryo development. Interestingly, a positive correlation between asthma and infertility has been shown [23,24], although the direct effect of budesonide on gestation and development has not been specifically investigated. Thus, the use of gastruloids as a platform to test drug toxicity effects could be crucial.

In light of these considerations, the identification of a novel budesonide analogue, i.e., CHD-030498, which shows effects similar to budesonide but at a significant lower concentration, may help to develop a potentially less toxic drug for other applications.

Budesonide has been shown to reduce extracellular collagen accumulation (fibrosis), stabilize the cell–cell interactions in primary tumors and decrease the appearance of lung metastasis in orthotopic breast cancer xenograft [17]. Thus, it would be interesting in the future to assess the effect of CHD-030498 in pathological contexts, including cancer progression and fibrotic diseases.

In conclusion, our findings highlight CHD-030498 as a potent inhibitor of cellular plasticity both on 2D systems and on the processes that are dependent on the initial formation of 3D cell aggregates, as gastruloids, acting in a lower concentration range. Changing the budesonide chemical structure has allowed identifying of a new bioactive compound with higher potency that, if proven valid, in the future might represent a potential novel drug candidate for the treatment of a broad spectrum of diseases in which cell fate transition and fibrosis are involved, including metastatic cancer. 

## Figures and Tables

**Table 1:** Sequences of the primers used for qPCR analysis.

Gene	Primer Forward	Primer Reverse
*N-Cad*	AGCGCAGTCTTACCGAAGG	TCGCTGCTTTCATACTGAACTTT
*Mmp2*	TTCCCTAAGCTCATCGCAGACT	CACGCTCTTGAGACTTTGGTTCT
*T*	GAACCTCGGATTCACATCGT	TTCTTTGGCATCAAGGAAGG

## Data Availability

No new data were created.

## Supplementary Materials

The following supporting information can be downloaded at: https://www.mdpi.com/article/10.3390/pharmaceutics15071897/s1, Figure S1. Effect of BA-GCs on ESCs proliferation. Figure S2. Effect of BA-GCs on mesenchymal-to-embryonic stem cell transition (MesT). Figure S3. Effects of proline on the proliferation of GR knock-down cells. Figure S4. Time-course and dose-response effect of CHD-030498 on pluripotencyexit. Figure S5. Dose-response activity of CHD-030498 on gastruloid development.
