# Supplementary material for: Budesonide Analogues Preserve Stem Cell Pluripotency and Delay 3D Gastruloid Development
Source: Pharmaceutics. 2023 Jul 6;15(7):1897. doi: 10.3390/pharmaceutics15071897 (PMC10383393; doi:10.3390/pharmaceutics15071897)
Supplement: Supplementary file 1 [file pharmaceutics-15-01897-s001.zip › pharmaceutics-2445655-supplementary.pdf]

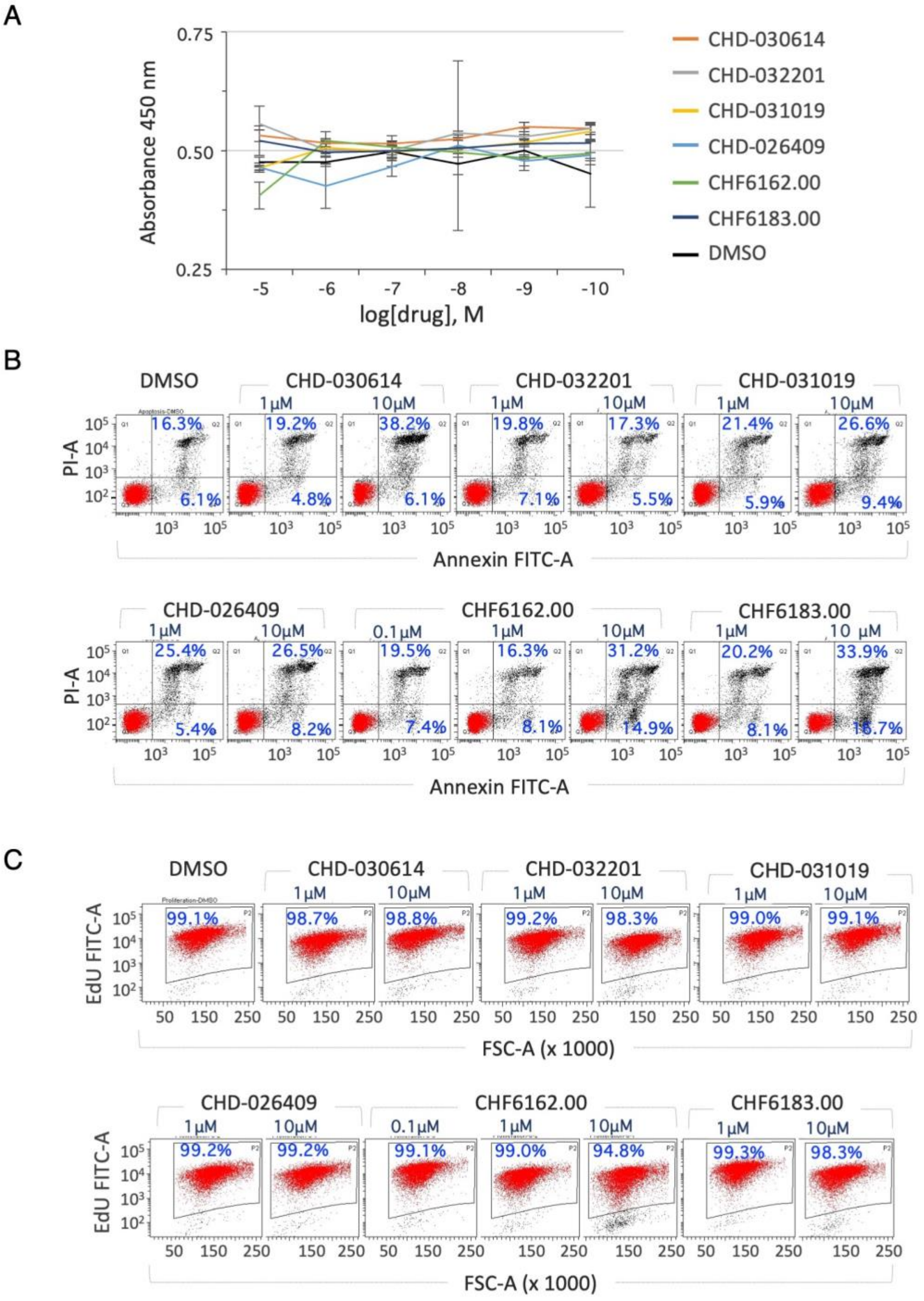

Supplementary Figure S1. Effect of BA-GCs on ESCs proliferation. (A) Effect of BA-GCs on ESC survival at the following concentrations: 10^{-10}, 10^{-9}, 10^{-8}, 10^{-7}, 10^{-6}, 10^{-5} M, for 48 hrs, by CCK-8 assay. Data are shown as mean ± SD (n = 3; $p \leq 0.01$). (B) FACS analysis of apoptotic cells. Representative dot plot of Annexin V/PI staining in ESCs treated with DMSO as Control, and treated with the different BA-GCs used at the indicated concentrations. The fraction (%) of Annexin V+ (bottom right) and Annexin V+/ PI+ (top right) cells are shown. Annexin V is conjugated with FITC. (C) FACS analysis of EdU incorporation. Representative dot plot of EdU incorporation in ESCs treated with DMSO as Control, and treated with BA-GCs at the indicated concentrations. The fraction (%) of cells that incorporated EdU is shown.

A

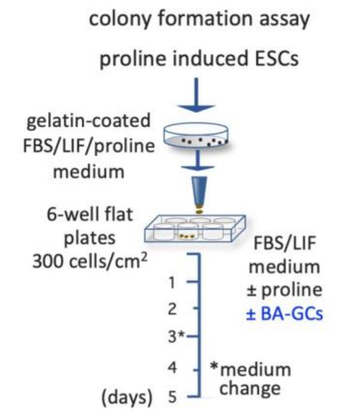

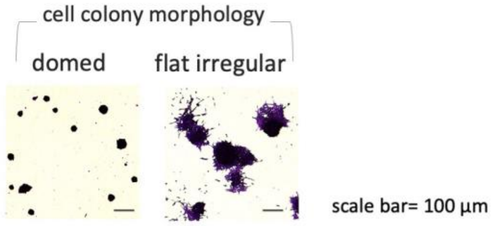

	% domed colonies (± SD)
DMSO	67±2
proline	33±1.7

B

% domed* colonies (± SD)

	compound [µM]	10	1
proline plus	budesonide	74±0.9	40±1.2
	fluticasone	36±1.6	NE
	CHD-030614	79±1.9	54±2
	CHD-032201	89±1.2	35±0.9
	CHD-031019	52±0.7	40±1.3
	CHD-026409	40±2.3	40±1.8
	CHD-030498	NG	70±3
	CHF6162.00	NG	40±1.6
	CHF6183.00	67±0.8	45±0.7

*MesT induction NG= No Growth NE= No Effect

Supplementary Figure S2. Effect of BA-GCs on mesenchymal-to-embryonic stem cell transition (MesT). (A) Schematic representation of the experimental procedure (left). Representative photomicrographs of domed- and flat-shaped cell colonies (top right) and quantification of the fraction (%) of domed-shaped cell colonies generated from proline-induced mesenchymal-like cells treated ± proline (250 µM) (bottom right). (B) Table showing the fraction (%) of domed-shaped cell colonies (MesT induction), generated from proline-induced mesenchymal-like cells treated with proline (250 µM) ± BA-GCs at the indicated concentrations. Budesonide and fluticasone were used as a positive (MesT inducer) and negative (inactive) Control. Data are shown as mean ± SD (n=3).

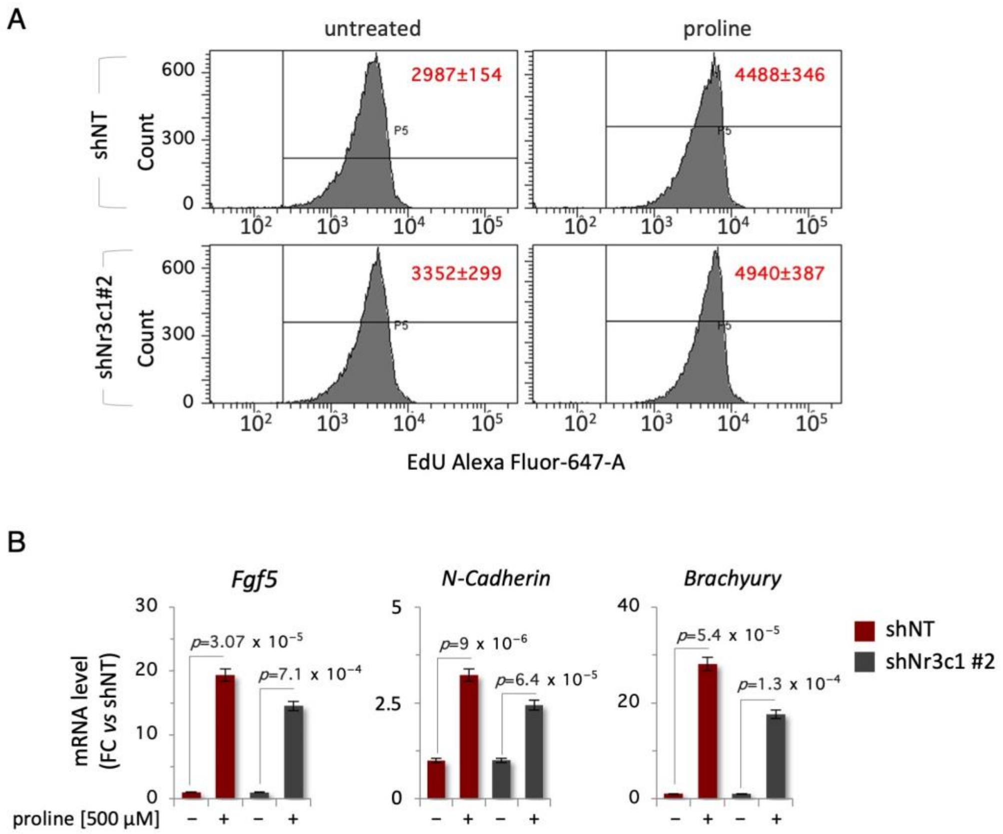

Supplementary Figure S3. Effects of proline on the proliferation of GR knock-down cells. (A) FACS analysis of proliferating cells. Representative histograms of EdU incorporation in shNT and shNr3c1#2 ESCs ± proline (500 µM) are shown. The mean intensity of EdU incorporation is shown. Data are mean ± SEM (n=3). (B) Effect of GR on the expression of esMT marker genes. qPCR analysis of Fibroblast Growth Factor 5 (*Fgf5*), Neural Cadherin (*N-Cadherin*) and Brachyury gene expression. Values are expressed as a fold-change *versus* shNT ESCs, after normalization to *Gapdh* and are mean ± SEM (n=3).

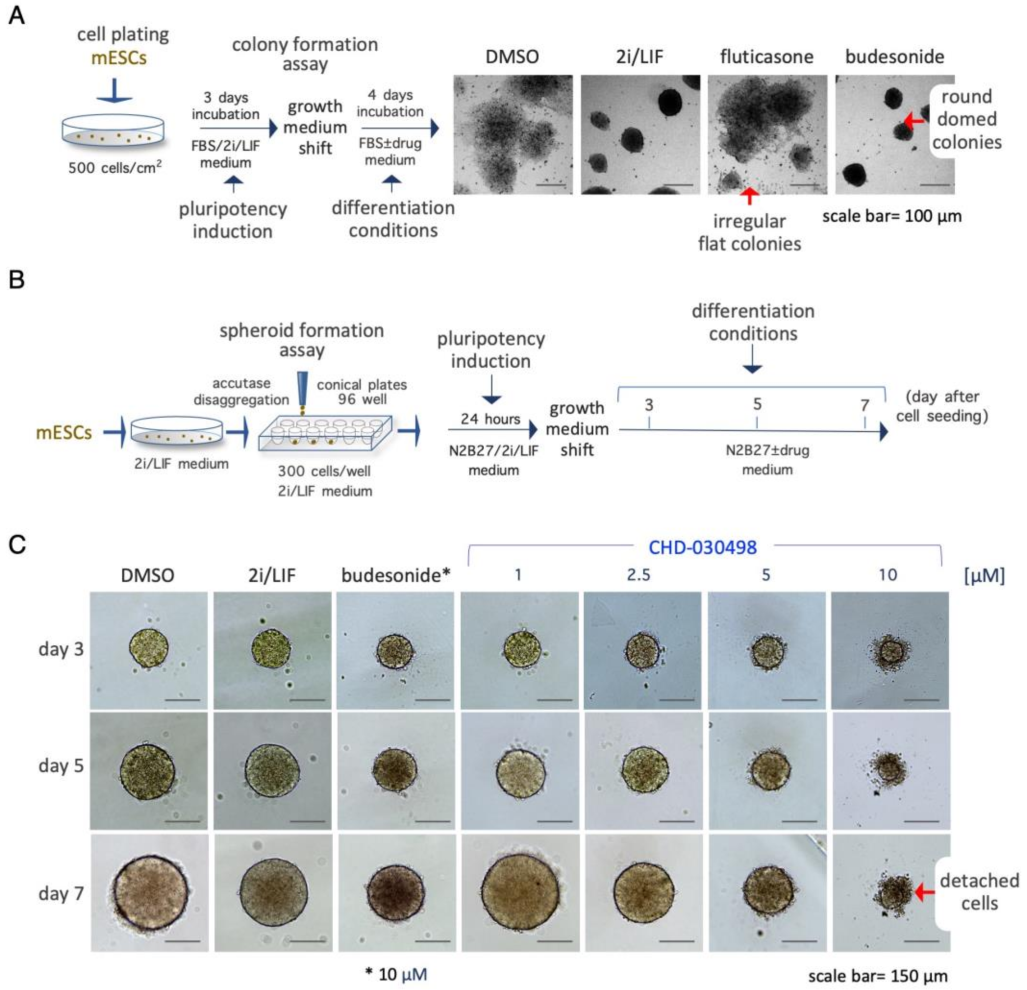

Supplementary Figure S4. Time-course and dose-response effect of CHD-030498 on pluripotency exit. (A) Schematic representation of the pluripotency exit procedure (left). ESCs were plated (500 cells/cm^2) in DMEM/FBS/2i/LIF medium for 3 days, and then shifted to DMEM/FBS medium supplemented with DMSO, 2i/LIF, fluticasone, or budesonide. After 4 days of incubation the cell colonies were imaged (bright-field images; right). Scale bar, 100 µm. (B) Schematic representation of the experimental procedure. ESCs (2i/LIF) were FACS sorted (300 cells/well) on 96-well ultra-low conical plates and incubated in N2B27 ± DMSO, 2i+LIF or CHD-030498 (from 1 to 10 µM). (C) Representative bright-field images of spheroids obtained as described in (B). Images were captured for each condition at day 3, 5 and 7. and measured. Scale bar, 150 µm.

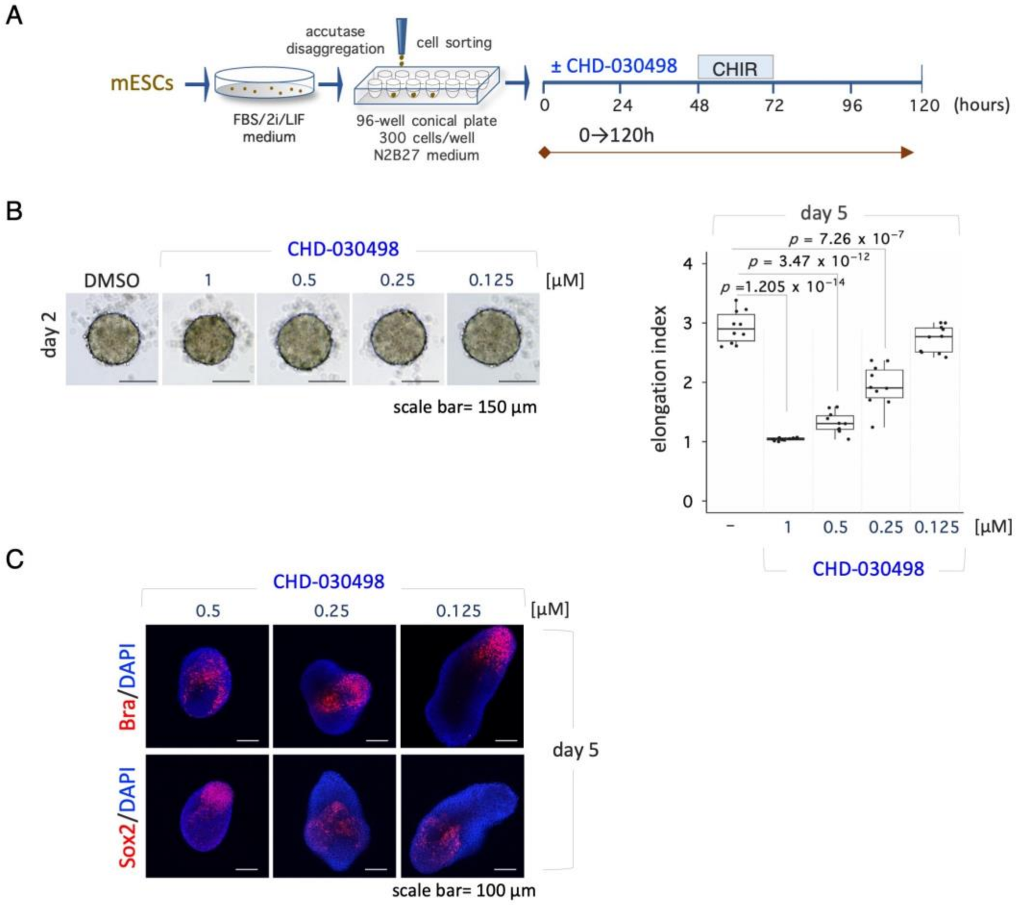

Supplementary Figure S5. Dose-response activity of CHD-030498 on gastruloid development. (A) Schematic representation of the experimental design. Naïve pluripotent (2i/LIF) ESCs were FACS sorted (300 cells/well) on 96-well ultra-low conical plates in N2B27 ± CHD-030498 added at 1, 0.5, 0.25 and 0.125 µM, from t0 to t120 hrs. (B) Representative bright-field images (left) and boxplot diagrams of gastruloid elongation index (right) of 2-day-old aggregates treated with DMSO (control), or CHD-030498 at 1, 0.5, 0.25 and 0.125 µM (n = 3; 20 gastruloids/condition). Scale bar, 150 µm. (C) Immunofluorescence analysis of differentiation (Brachyury, Sox2) markers. Representative confocal images of 5-day-old aggregates/gastruloids treated with CHD-030498 added at the indicated concentrations. Nuclei were counterstained with DAPI (blue). Scale bar, 100 µm.
